# A literature review of cancer diagnostic tests and treatments in adults with intellectual disability

**DOI:** 10.12688/hrbopenres.14164.1

**Published:** 2025-06-13

**Authors:** Kennedy Smihula, Mikayla Danon, Shauna Walsh, Martin McMahon, Louise Lynch

**Affiliations:** 1 College of Nursing, The Pennsylvania State University, University Park, Pennsylvania, USA; 2 Trinity College Dublin School of Nursing and Midwifery, Dublin, Leinster, Ireland

**Keywords:** Autonomy, Cancer, Intellectual Disability, Screening, Treatments

## Abstract

**Background:**

Adults with intellectual disabilities have significantly lower rates of routine cancer screening and cancer is often diagnosed at more advanced stages. Some studies specifically highlight gaps that exist in national screening programmes for cancers such as breast, cervical and colorectal. Evidence in the intellectual disability population points towards factors such as limited screening education, distrust in healthcare providers, and inability to provide consent, leading to limited uptake of available screening programmes. While there are many contributing factors to these inequalities, changes in individuals' health status may go unrecognised for significantly longer because of their intellectual disability.

**Methods:**

Five electronic databases were systematically searched: Cinahl Ultimate, Medline, PsycINFO, PubMed, and Web of Science. To provide the most accurate search, search functionality and keywords were used and adjusted for each database. Thematic analysis was completed using the Braun and Clark Six Step process.

**Results:**

Four main themes emerged: Prevention, education, adaptation, and autonomy. Prevention encompassed individuals receiving regular screening and barriers that prevented access. Educational tools that explained the importance of screening reduced feelings of stress and anxiety. Case studies illustrated how specific treatment plans were adapted for patients with intellectual disabilities. Autonomy and honesty were themes throughout many studies, in terms of treatment, education, and diagnostics. It was determined that patients should be involved in decision making and be aware of their cancer diagnosis unless there are contra-indications.

**Conclusion:**

Adults with intellectual disabilities face considerable barriers when accessing cancer diagnosis and treatment. Barriers, including living conditions, communication difficulties and age, contributed to later cancer diagnosis and worse outcomes, compared to the general population. The successful use of education and tailored treatments were enabling factors.

## Introduction

In recent years improvements in cancer care and survival in the general population have been observed Loud & Murphy (2017). Studies have emphasised the importance of regular screening and the role that this plays in the early detection of cancer ( Ding *et al.,* 2022; Miller, 2013; Whitaker, 2020). Advancements in treatment options, as well as secondary prevention and early detection, are leading to better outcomes and decreased cancer mortality rates in the general population ( Ding *et al.*, 2022; Gini *et al.*, 2020; Jemal *et al.*, 2010). Despite these recent developments in treatment, and the implementation of national cancer screening programmes, large gaps exist in diagnostic and treatment options between people with intellectual disabilities and the general population ( Cuypers *et al*., 2024; Mahar *et al*., 2024).

Adults with intellectual disabilities have significantly lower rates of routine cancer screening and cancer is often diagnosed at more advanced stages ( Horsbøl *et al.,* 2023; Kiani *et al*., 2014). Some studies specifically highlight gaps that exist in national screening programmes for cancers such as breast, cervical and colorectal ( Satgé *et al.*, 2023; Sullivan *et al.*, 2003; Swaine *et al.*, 2014). Evidence in the intellectual disability population points towards factors such as limited screening education, distrust in healthcare providers, and inability to provide consent, leading to limited uptake of available screening programmes and there appear to be misassumptions on women with intellectual disability not requiring cervical screening ( Power *et al.*, 2024; Swaine *et al*., 2014; Weise *et al*., 2024; Xu *et al*., 2017). While there are many contributing factors to these inequalities, changes in individuals' health status may go unrecognised for significantly longer because of their intellectual disability ( Kiani *et al*., 2014). In some cases, symptoms suggestive of cancer and cancer itself go undetected, and cancers are identified at a late stage or as a cause of death in individuals with intellectual disabilities ( Heslop *et al.*, 2022; Mahar *et al*., 2024). Communicating symptoms of cancer may be difficult for individuals with an intellectual disability, and those around them may not notice signs of illness, both reasons which may contribute to a late-stage cancer diagnosis ( Satgé *et al.*, 2014). Ultimately this phenomenon, known as diagnostic overshadowing, may contribute to a decreased quality of life and the unnecessary progression of cancer, resulting in premature mortality ( Mason & Scior, 2004; McMahon *et al.*, 2024).

Although cancer is being diagnosed at more advanced stages in adults with intellectual disabilities, the evidence does not show that diagnosis occurs later in life ( Mahar *et al.,* 2024; Satgé *et al.*, 2014). In a study conducted by Satgé and colleagues, the age at which individuals with intellectual disabilities were being diagnosed with cancer was younger than the recommended screening age for the general population with Heslop *et al*. (2022) and Mahar *et al.* (2024) reporting similar findings in England and Canada respectively. This may be an indication that adults with intellectual disabilities are developing cancer earlier in adulthood, and screening protocols may need to be tailored to this. Recent evidence has shown that individuals with intellectual disabilities were 1.6 times more likely to be diagnosed with Stage IV breast cancer and 1.44 times more likely to be diagnosed with Stage IV colorectal cancer ( Mahar *et al*., 2024). While the reasons why they are developing late-stage cancer at earlier ages have not yet been established, studies suggest that lowering screening ages for people with intellectual disabilities could be beneficial for earlier diagnosis. The prognosis for cancer patients is dependent on the cancer stage at diagnosis, so a later diagnosis in patients with intellectual disabilities could be a key factor accounting for differences in mortality rates.

There is limited research that focuses on treatment options for individuals with intellectual disabilities diagnosed with cancer. Much of the published evidence are case studies based on one individual's cancer experience with no synthesis of the published evidence available ( Brown, 2011; Enomoto *et al*., 2015). In terms of diagnostic tools, while individuals with intellectual disabilities may utilise the same tools as the general population, tools such as mammography, Papanicolaou tests (pap smears), and testicular examinations, remain under-utilised by the intellectual disability population ( Satge *et al.*, 2023). The existing gaps in cancer diagnosis may be from patients not receiving regular screening ( Chan *et al.*, 2022; Sullivan *et al.*, 2003).

Although the exploration of treatments in case studies are specific to individual patients, these treatments may set a precedent that tailored treatment plans are required for the specific needs of this vulnerable population ( Brown, 2011; Delany *et al*., 2023; Satgé *et al*., 2014; Sleijfer *et al*., 1996). Consequently, the aim of this literature review is to explore and synthesise information relating to cancer diagnostic approaches and treatment options for adults with intellectual disabilities and examine the barriers that prevent adults with intellectual disabilities from accessing both diagnostic tools and treatments.

## Methods

This literature review was designed to understand the experiences of adults with intellectual disabilities undergoing cancer diagnostic tests and treatments. Literature reviews provide a thorough critique and examination of all the evidence available on the topic and facilitate a descriptive analysis and meaningful synthesis of the current available research ( Colquhoun *et al.*, 2014)

### 1. Research question

The PICOS (Population, Intervention, Comparison, Outcome, Study Type) framework was used to frame the research question and search concepts for this review. The research question to be addressed is:

*‘What are the barriers or enablers to accessing cancer diagnostics and treatment for adults with intellectual disability?’*

This will be achieved through two objectives:

- Explore and synthesise information relating to cancer diagnostic approaches and treatment options for adults with intellectual disabilities
- Examine barriers and enablers to accessing cancer diagnostic tools and treatments for adults with intellectual disability.

### 2. Eligibility criteria

The eligibility criteria are summarised in Table 1.

**Table 1.** Literature review eligibility criteria.

	Include	Exclude
**Population**	- Adults with all levels of intellectual disability - Down syndrome and other syndromes (if intellectual disability presence confirmed)	- Children, teenagers under 18 years of age - Adults without intellectual disability
**Intervention/Exposure**	- Any diagnosis and treatment of cancer	- Studies with no specified focus on diagnosis or treatment of cancer
**Comparison**	- Individuals living in residential, community group homes, with family or independently	- None. All living circumstances included.
**Outcome**	- Cancer (any type)	- Studies reporting outcomes not related to cancer
**Study Characteristics**	- Observational (retrospective and observational) - Qualitative - Cohort studies - Randomised control trials	- Reviews - Editorials or opinion pieces - Book chapters
**Language**	- English	- All non-English languages

### 3. Search strategy

Five electronic databases were systematically searched: Cinahl Ultimate, Medline, PsycINFO, PubMed, and Web of Science. To provide the most accurate search, search functionality and keywords were used and adjusted accordingly for each database. Keywords included “intellectual disabilities”, “treatment”, “diagnosis”, and “cancer”. Boolean operators were employed, including “and”, “or”, along with “not”. Table 2 shows an example of the MEDLINE search string. Finally, citations were searched in relevant reviews about cancer treatments and screening for adults with an intellectual disability. All articles were extracted to Covidence which was used as the screening tool.

**Table 2.** An example of the MEDLINE search string.

Concept	Index	Keywords
**Concept 1:** Cancer	(MH "Cancer")	(Cancer OR oncology OR neoplasm OR malignant OR malig*)
**Concept 2:** Intellectual disability or learning disability	(MH "Intellectual Disability+") OR (MH "Learning Disabilities+")	((intellectual AND disabilit* OR 'mental retardation'/exp OR 'mental retardation' OR (mental AND ('retardation'/exp OR retardation)) OR 'learning'/ exp OR learning) AND disabilit* OR developmental) AND disabilit* OR 'learning disabilities'/exp OR 'learning disabilities' OR (('learning'/exp OR learning) AND disabilities)
**Concept 3:** Diagnosis	(MH “Diagnosis”)	(diagnos* OR screen* OR assess* OR evaluation OR detect* OR identif*)
**Concept 4:** Treatment	(MH “Therapeutics”)	(treat* OR therap* OR intervention OR management OR care OR "therapeutic approach" OR "clinical management")

### 4. Screening process

**Figure 1.**
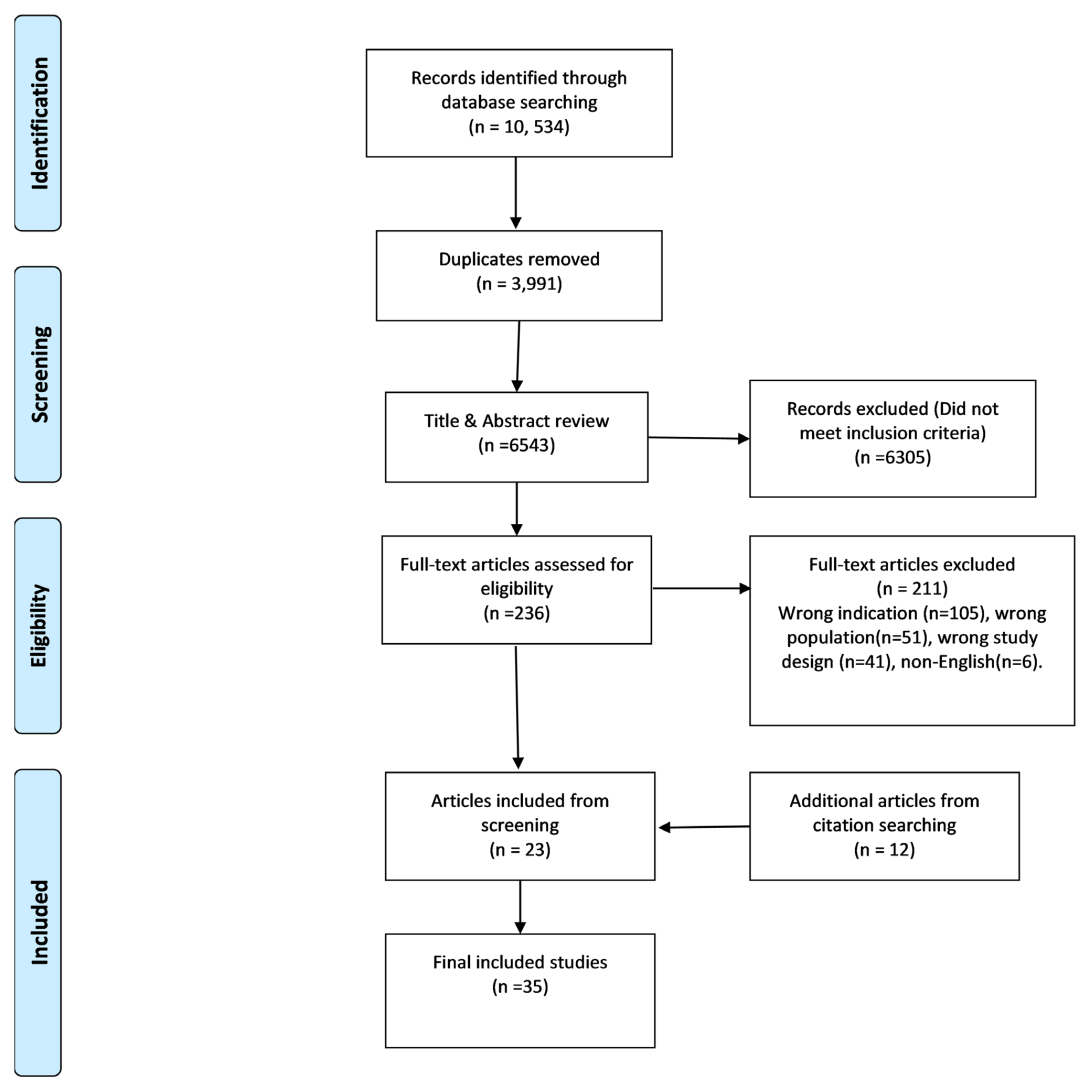
PRISMA diagram.

Figure 1 summarises the screening process in a PRISMA diagram. Two assessors [KS, MD] reviewed each of the articles. A third [LL or MMcM] adjudicated in cases of dispute. The initial search resulted in 10,534 publications with 3,991 duplicates removed. A title and abstract screening of 6,543 articles was completed. Then, 236 articles proceeded to full-text review. Finally, 23 articles were extracted, along with 12 articles from citation searching, resulting in a total of 35 articles for inclusion in the literature review.

### 5. Data extraction

Data was gathered from studies conducted worldwide from 1996 to 2024. The following information was extracted from each article: author, year, country, study aim, study design, sample size, age, gender, level of intellectual disability, diagnosis or treatment, measurement method, outcome, summary, conclusions, and any notes, see Table 2. All information was not available for each article.

### 6. Thematic analysis

A qualitative synthesis and thematic analysis was completed on the extracted articles using the Braun and Clark Six Step process, see Table 3 ( Braun & Clarke, 2006). Initially the researchers familiarised themselves with each article’s content by reading multiple times and making notes on each, to fully immerse themselves in the data. As initial patterns emerged, colour-coding was used to group similar codes together. Broad themes emerged as a result of this grouping. These broad themes were then refined further, analysing the codes that were contained in each theme until four broad themes remained. Some articles were grouped into multiple themes as they covered multiple topics.

**Table 3.** Six step thematic analysis process.

Step number	Process	Explanation
**1**	Data familiarisation	Complete data immersion
**2**	Generate initial codes	Topics, patterns of data
**3**	Search for themes	Broader theme identification
**4**	Review of themes	Theme refinement
**5**	Define and name themes	Categorise. Include sub-themes if required
**6**	Produce report	Complete write-up

( Braun & Clarke, 2006)

## Results

A total of 35 articles were included in the final review. After completing the Braun and Clark thematic analysis, the four overriding themes emerged i.e. Prevention, Education, Adaptation and Autonomy. Some overlap was observed in articles that covered multiple themes ( Armin *et al.,* 2022; Brown, 2011; Flynn *et al.,* 2016; Sleijfer *et al.,* 1996; Sullivan *et al.,* 2003). The number of people with intellectual disabilities represented in this review was 97,099. Studies were representative of a worldwide population; USA (n=7), UK (n=6), Canada (n=3), France, Australia and Japan (n=2), and Sweden, Denmark, Taiwan and The Netherlands (n=1). Overall, 14 articles investigated preventative measures of cancer screening, seven articles discussed education on cancer and screening for people with intellectual disabilities, seven articles reviewed cancer treatment options for patients with intellectual disabilities, and three articles discussed patient autonomy, see Table 4 in extended data. A summary of each theme’s results is provided. Screening was addressed in prevention and education. Treatments were covered by adaptation and autonomy.

### (a) Prevention

The importance of cancer screening as a preventative measure for people with intellectual disabilities due to noted higher risks of developing cancer and having difficulties with explaining their pain and symptoms, was discussed in 14 articles. Armin *et al.* (2022) found that social inequalities contributed as barriers to screening for adults with an intellectual disability while intellectual disability severity level was negatively associated with screening levels ( Horsbøl *et al.,* 2023). Additionally, this population were less likely to receive cancer screening in comparison to the general population due to unique barriers including living conditions, and distress and anxieties they experience may make them less likely to engage with screening ( Tuffrey-Wijne *et al.,* 2010). Furthermore, mobility, behavioural issues and a lack of mental capacity emerged as barriers for women with intellectual disability receiving cancer screening ( Bates & Triantafyllopoulou, 2018). Lower levels of cancer screening resulted in increased numbers of late-stage cancer diagnoses with higher levels of poor health outcomes ( Heslop *et al.,* 2022; Satgé *et al.,* 2014). A concern is that people with intellectual disabilities have presented with cancer at ages below those recommended for screening commencement for the general population ( Heslop *et al.,* 2022; Mahar *et al.,* 2024; Satgé *et al.,* 2023).

### (b) Education

Education about cancer screening and treatments was explored in seven articles. Various screening educational tools were used, including the ‘Mammography Preparedness Measure’ which assessed women’s readiness for Mammograms and a testicular cancer education programme which resulted in improved self-efficacy among men with intellectual disability ( Wang *et al.,* 2015; Wilson *et al.,* 2018). An American ‘My Health My Choice’ program demonstrated that education resulted in treatment options being offered that would otherwise not be presented ( Armin *et al.*, 2023). The American ‘Women Be Healthy 2’ program and DVDs as educational tools were positively received by adults with an intellectual disability and demonstrated an increase in knowledge of screening ( Greenwood *et al.,* 2014; Swaine *et al.,* 2014). 

### (c) Adaptation

The modification of treatments to make them more amenable to patients with intellectual disability was the third theme that emerged in this review. Specific cancer treatments for patients with intellectual disability were detailed in seven articles. Adapted chemotherapy was the focus of three articles, where the successful adjustment of treatments to meet the specific needs of the patients resulted in positive outcomes ( Delany *et al.,* 2023; Enomoto *et al.*, 2015; Sleijfer *et al.,* 1996). An article with a focus on radiation therapy showed that anaesthesia could be used to improve treatment tolerance ( Gandhidasan *et al.*, 2020). A standard approach to cancer surgery for those with and without intellectual disabilities showed that similar results were achievable ( Ishimaru *et al*., 2022). A study which investigated pain management for cancer patients found that people with an intellectual disability were less likely to receive opioids, compared to their peers in the general population ( Segerlantz *et al.,* 2019). The authors proposed that this lack of treatment was due to communication challenges rather than the absence of pain. Concerningly, a case study on a stem transplant for a patient with intellectual disability who lacked the understanding of the procedure, resulted in premature death and unwarranted suffering ( Brown, 2011). Some overlap was observed within studies where other treatments were also incorporated. 

### (d) Autonomy

Autonomy, where patients with an intellectual disability were able to decide on their own treatment options was the final theme. The three studies that investigated patient autonomy explored the unique role of care partners and their roles in supporting this population in health matters. More positive outcomes and meaningful engagement were seen with the proactive engagement of the patient in their own care while not updating the patient on their condition resulted in needless distress and an early death ( Brown, 2011; Flynn *et al.,* 2016). Studies also discussed the principle of “truth-telling” which is when medical providers and care partners determine how much, if at all they decide to explain to the patient about their diagnoses ( Bernal & Tuffrey-Wijne, 2008). Finally, patient autonomy highlighted the ethical debates that surround consent and patients with an intellectual disability ( Brown, 2011; Bernal & Tuffrey-Wijne, 2008; Flynn *et al.,* 2016). 

## Discussion

This literature review examines the cancer diagnostic approaches and treatments available for adults with intellectual disability and any barriers or enablers associated with them. Results identified the importance of tailored education and application to cancer screening, diagnosis and treatment, and more involvement of adults with intellectual disability in decisions about their own care, on positive impacts on their cancer outcomes. Unique barriers to screening access were identified.

Cancer is one of the leading causes of death worldwide, but treatments have high success rates if it is diagnosed at an early stage ( Bates & Triantafyllopoulou, 2018). By utilising secondary prevention, i.e. cancer screening and regular health checkups to diagnose cancer while it is still asymptomatic, cancer may be diagnosed at an early stage while it is still possible to have positive health outcomes ( Ouellette‐Kuntz *et al.*, 2015a). Nevertheless, individuals with intellectual disabilities may be unable to recognise signs and symptoms suggestive of cancer or be able to communicate their symptoms to a caregiver, and the caregiver may be unable to recognise symptoms which could make them more easily overlooked unless regular checks occur ( Ouellette‐Kuntz *et al.*, 2015a).

Cancer screening uptake is much lower in patients with intellectual disability than those without. For example, women with intellectual disabilities are five times more likely to never be screened for cancer than the general population and a lower rate of mammograms is observed in women with intellectual disability ( Ishimaru *et al.,* 2022; Lai *et al.,* 2014). A Taiwanese study reported that the breast cancer screening rate for women with intellectual disabilities was 4.3% compared to 12% of women in the general population, while pap smear rates were 4.8% compared to 28.8% in the general population ( Yen *et al.,* 2014). Similarly, men with intellectual disabilities had lower rates of cancer screening. A Canadian routine data-based study on colorectal cancer (n=807,583) reported that 18.3% of intellectual disability participants received a faecal occult blood test in the previous two years, while 32% were up to date with their colorectal screening, compared to the general population at 26.4% for screening in the last two years and 47.2% for being up to date with their screening ( Ouellette-Kuntz *et al.,* 2015b). Although the average lifespan of people with intellectual disability has increased, as theoretically has the risk for cancer developing increased, they are still not receiving equitable screening to the general population ( Flynn *et al.,* 2016; Lai *et al*., 2014; Mahar *et al.*, 2024; Satgé *et al.*, 2014; Wilkinson *et al.,* 2011).

People with intellectual disability may not receive necessary cancer screening due to a unique set of barriers ( Bates & Triantafyllopoulou, 2018; Delany *et al.,* 2023; Ouellette-Kuntz *et al.,* 2015b). A key risk factor for cancer is ageing, and screening for cancer detection is recommended at specific ages. Historically, the intellectual disability population had shorter lifespans, and many would not reach the age when preventative measures commenced and therefore were excluded from standard screening ( Flynn *et al.,* 2016; Lai *et al*., 2014; Mahar *et al.*, 2024; Satgé *et al.*, 2014; Wilkinson *et al.,* 2011).

Elevated rates of late diagnosis are reported for people with intellectual disabilities compared to the general population. Studies indicate that patients with intellectual disability were more likely to detect breast, colorectal, and lung cancer at stages III and IV rather than stages I or II ( Mahar *et al*., 2024; Satgé *et al.*, 2023). These late-stage diagnoses of cancers meant that only limited treatments were available, and a high mortality rate occurred. Furthermore, people with intellectual disability have an onset of tumours at earlier ages than that of the general population, so if normal screening timelines are followed it may be too late for a timely diagnosis ( Heslop *et al*., 2022; Mahar *et al.,* 2024).

Living circumstances was another barrier to screening access ( Sullivan *et al*., 2003; Xu *et al.,* 2017). A protective factor was seen for those who resided in a group home or medical facilities, where they were more likely to receive cancer screening due to established policies and procedures ( Xu *et al.,* 2017). In contrast, others who lived in institutionalised care facilities may have a higher level of intellectual disability and the facility may have less resources, causing them to be screened less ( Armin *et al*., 2022; Sullivan *et al.,* 2003). Additionally, if a patient lives alone in a rural environment, transportation to screenings may be difficult, often leading to less screening attendance ( Yen *et al*., 2014).

Cognitive deficits associated with intellectual disability may make understanding cancer screening procedures or communicating with relevant specialists more difficult, which can heighten stress and anxiety surrounding procedures, resulting in their cancellation ( Bates & Triantafyllopoulou, 2018; Horsbøl *et al.*, 2023; Wilkinson *et al*., 2011). A need for education on cancer screening was identified as critically important, in regard to enabling a person with an intellectual disability to engage with screening. Many individuals with intellectual disabilities are not educated about the need for cancer screening or the processes involved, and often do not feel prepared to attend screening, which reduces uptake ( Wilkinson *et al*., 2011). A lack of education and preparation has contributed to large gaps in cancer screening ( Ishimaru *et al*., 2022). In a study conducted with females with intellectual disability, patients expressed feelings of confusion, and felt they could not communicate their emotions with their providers and that their doctors did not understand the extent of their confusion ( Wilkinson *et al*., 2011). Furthermore, patients felt they were misinformed or given inadequate information about screening procedures resulting in inadequate preparation. Studies revealed that health professionals should take the time to explain to individuals with intellectual disabilities the specific details of mammograms; where they occur, how long they take and describe the experience using easy-read material ( Sullivan *et al.*, 2003; Wilkinson *et al.*, 2011). It was also suggested that doctors provide additional support options such as a video for clarity ( Wilkinson *et al.,* 2011).

Studies have used different assessment tools such as the Mammography Preparedness Measure (MPM) to address perceived gaps in knowledge of cancer screening for those with intellectual disabilities ( Greenwood *et al.*, 2014; Wang *et al*., 2015). The MPM is a widely used, validated tool to assess if women are emotionally and physically prepared to undergo a mammogram ( Greenwood *et al.*, 2014; Wang *et al*., 2015). This is not only useful in determining whether individuals warrant education on mammograms but is also used to evaluate the success of various forms of mammography education by performing the MPM before and after the education.

Diverse educational tools were employed to address patients' screening knowledge. In the US, researchers used a DVD of an individual with an intellectual disability who overcame barriers during mammography screening, to reduce feelings of fear, and unpreparedness ( Greenwood *et al*., 2014). The DVD was a successful form of increasing preparation for mammography and highlighted the positive impact of presenting a relatable visual representation. Other tools integrated both breast and cervical cancer knowledge. The ‘Women Be Healthy 2’ was an 11-week programme which provided cervical and breast screening information, as well as preparation for screening ( Swaine *et al*., 2014). Results indicated that ‘Women Be Healthy 2’ was effective in educating women with intellectual disabilities about cancer screening. A study called ‘My Health, My Choice’ also focussed on providing cervical and breast cancer screening education to Native American Women with intellectual disabilities and taught them to be able to identify cancer, and to understand the screening processes, utilising hands-on activities to help the women manage their anxiety ( Armin *et al*., 2022). This program demonstrated an increase in cancer knowledge and screening among participants, demonstrating a link between higher levels of education and higher levels of screening engagement.

Studies also explored education surrounding testicular cancer in individuals with intellectual disabilities. An educational programme about testing testicular cancer awareness was developed which utilised a video, anatomical visual aids, and a computer programme to increase individuals' understanding of cancer, and to help them be able to recognize the signs and symptoms sooner ( Wilson *et al.*, 2018). Both the educational programme and the information leaflet were determined to have a positive effect on individuals' knowledge and confidence in performing a testicular self-examination.

While education is an important foundation for supporting patients with intellectual disability, it is crucial that this is provided in conjunction with adaptations for cancer treatments ( Armin *et al.,* 2023). Healthcare providers should tailor care to each individual's unique cognitive and emotional needs. A variety of treatments were explored in this review. Chemotherapy is a major treatment of cancer, however because of its strength and negative side effects, providers will not always offer this as an option for patients with intellectual disabilities ( Delany *et al*., 2023). In a case study with a patient with testicular cancer, where it was felt that standard chemotherapy would not be tolerated by the patient, a modified regime resulted in the successful treatment of the cancer and he was able to return to normal daily activities. The study concluded that following evidence-based practice is not always the best route of cancer care for individuals with intellectual disabilities, and that health care providers need to take time to make the necessary adjustments to care. Another example of successful chemotherapy was in a patient with tongue cancer where alternative treatments resulted in the successful eradication of the cancer ( Enomoto *et al*., 2015). These case studies demonstrated that individuals with intellectual disabilities should be considered for chemotherapy treatments, and providers should tailor treatment and care to their specific needs.

 Radiation was another integral part of cancer treatment that was successfully adapted and employed in patients with intellectual disabilities. A study reported on the successful effective use of outpatient anaesthesia and delivery of radiation therapy in patients with intellectual disabilities who had early-stage lung cancer, for whom surgery was not an option, where the care team individualised care based on the unique needs of the patients ( Gandhidasan *et al*., 2020). Surgery was another form of cancer treatment explored in individuals with intellectual disabilities. There are ethical debates about men with testicular cancer undergoing radical inguinal orchidectomy for both diagnosis and treatment, but the cure rate can be increased by finding an appropriate balance of therapy ( Hafeez *et al*., 2015). A study evaluating surgery in individuals with severe motor and intellectual disabilities, showed seven patients who had cancer surgery all survived and reported satisfactory outcomes ( Ishimaru *et al*., 2022).

 Ethical principles play a large role in the type of cancer treatment that individuals with intellectual disabilities receive. In the case of a patient who received a stem cell transplant, an ethical debate ensued about whether the patients with intellectual disabilities should receive a difficult treatment he did not understand ( Brown, 2011). After receiving the treatment, the patient developed complications and died soon after. This case study largely focused on treatment ethics, however this known risky therapy was not proven to be successful because of the complications the patient experienced, independent of the presence of an intellectual disability. Similarly, studies explored debates regarding pain management and prescription drugs being used in the treatment of individuals with intellectual disabilities ( Axmon *et al.,* 2018; El-Tallawy *et al.,* 2023; Millard & de Knegt, 2019; Segerlantz *et al.*, 2019). Older adults with cancer and intellectual disabilities were less likely to receive prescription pain medications then their counterparts resulting in the under-management of pain and a reduction in quality of life ( Segerlantz *et al.*, 2019).

The ethical debate of treating cancer patients with intellectual disabilities continues into the theme of autonomy. Having an intellectual disability may inhibit an individual's understanding of a plethora of topics which may further alter how decisions are made ( Hafeez *et al.,* 2015). Nonetheless, the intellectual disability community has a long history of debating how to handle decision-making and notifying patients with intellectual disabilities of different diagnoses, especially when it comes to cancer, complicated by individual country legal frameworks and capacity ( Delany *et al.*, 2023; Kiani *et al.*, 2014; Tuffrey-Wijne *et al.*, 2010). Often people with intellectual disabilities have difficulties being independent and may have carers who have influence over their medical, educational, and financial decisions ( Kiani *et al.*, 2014). A lack of truth-telling stems from wanting to protect the patient from excess anxiety and distress, though it may limit the patient’s autonomy. However, a lack of evidence was seen about distress after cancer diagnosis in patients with intellectual disability, implying that these patients should be entitled to honesty when it comes to their care and making medical decisions ( Bernal & Tuffrey-Wijne, 2008; Flynn *et al.,* 2016). Furthermore, medical professionals participated in non- “truth-telling” due to feelings of discomfort when working with this population, and they conferred solely with care partners about the patient's condition rather than with the patient themselves ( Flynn *et al.,* 2016; Tuffrey-Wijne *et al.,* 2010). Studies also found evidence where carers have asked for the professionals to discuss diagnosis with the patients and the professionals refused. Brown explains an ethical dilemma where a patient who was unable to consent for themselves. The best practice determined by the medical team for the patient was to undergo aggressive treatment despite doubts raised by nursing staff ( Brown, 2011). It was reported that the patient passed away from pneumonia within a year of treatment, which brings into question the overall suitability of the treatment. Informed consent is a demonstrated successful consent method for patients with intellectual disabilities and should be used as part of a standard protocol. These findings may serve as a guide for healthcare professionals in providing diagnostic and treatment options, educating their patients, and communicating with patients with intellectual disabilities about their illness.

Notwithstanding these findings, this literature review has several limitations including an over reliance on case studies with the heterogeneity of study designs making synthesis more challenging. Additionally, cancer diagnosis and treatment may have a geographical influence which may impact study findings. Equally, the subjective nature of thematic analysis may affect the interpretation and applicability of findings. Lastly, aged results are reported due to a dearth of recent evidence.

## Conclusion

 This literature review highlights major differences in treatment and diagnosis needs in cancer patients with intellectual disabilities and their general population peers. Unique barriers to screening including living conditions, age, life span and communication difficulties, contributed to later diagnosis and worse cancer outcomes.

In summary, this literature review shows that people with intellectual disability should be provided with more education on cancer screening and treatment options, should have access to screening at earlier ages, should be provided with more tailored treatment options and their autonomy should be given due consideration.

## Data Availability

No data were associated with this article Harvard Dataverse- **Replication Data for: Table 4: Article extraction summary information** https://doi.org/10.7910/DVN/VD23QF ( Lynch, 2025) Data available under CC0 1.0 licence
