# A literature review of cancer diagnostic tests and treatments in adults with intellectual disability

**DOI:** 10.12688/hrbopenres.14164.2

**Published:** 2026-02-24

**Authors:** Kennedy Smihula, Mikayla Danon, Shauna Walsh, Martin McMahon, Louise Lynch

**Affiliations:** 1 College of Nursing, The Pennsylvania State University, University Park, Pennsylvania, USA; 2 Trinity College Dublin School of Nursing and Midwifery, Dublin, Leinster, Ireland

**Keywords:** Autonomy, Cancer, Intellectual Disability, Screening, Treatments

## Abstract

**Background:**

Adults with intellectual disabilities have significantly lower rates of routine cancer screening and cancer is often diagnosed at more advanced stages. Some studies highlight gaps that exist in national screening programmes for cancers such as breast, cervical and colorectal. Evidence in the intellectual disability population points towards factors such as limited screening education, distrust in healthcare providers, and challenges in providing consent, leading to limited uptake of screening programmes. While there are many contributing factors to these inequalities, changes in individuals' health status may go unrecognised for longer because of their intellectual disability. The aim of this literature review is to explore cancer diagnostic approaches and treatment options for adults with intellectual disability and examine barriers that prevent adults with intellectual disability from accessing diagnostic tools and treatments.

**Methods:**

Five electronic databases were systematically searched: Cinahl Ultimate, Medline, PsycINFO, PubMed, and Web of Science. Thematic analysis was completed using the Braun and Clark Six Step process.

**Results:**

Four main themes emerged from 28 included studies: Prevention, education, adaptation, and autonomy. Prevention encompassed individuals receiving regular screening and barriers that prevented access. Educational tools that explained the importance of screening reduced feelings of stress and anxiety. Case studies illustrated how specific treatment plans were adapted for patients with intellectual disability. Autonomy and honesty were themes throughout many studies, in terms of treatment, education, and diagnostics. It was determined that patients should be involved in decision making and be aware of their cancer diagnosis unless there are contra-indications.

**Conclusion:**

Adults with intellectual disability face considerable barriers when accessing cancer diagnosis and treatment. Barriers, including living conditions, communication difficulties and age, contributed to later cancer diagnosis and worse outcomes, compared to the general population. The successful use of education and tailored treatments were enabling factors.

## Introduction

In recent years improvements in cancer care and survival in the general population have been observed Loud & Murphy (2017). Studies have emphasised the importance of regular screening and the role that this plays in the early detection of cancer ( Ding *et al.*, 2022; Miller, 2013; Whitaker, 2020). Advancements in treatment options, as well as secondary prevention and early detection, are leading to better outcomes and decreased cancer mortality rates in the general population ( Ding *et al.*, 2022; Gini *et al.*, 2020; Jemal *et al.*, 2010). Despite these recent developments in treatment, and the implementation of national cancer screening programmes, large gaps exist in diagnostic and treatment options between people with intellectual disability and the general population ( Cuypers *et al.*, 2024; Mahar *et al.*, 2024).

Adults with intellectual disability have significantly lower rates of routine cancer screening and cancer is often diagnosed at more advanced stages ( Horsbøl *et al.*, 2023; Kiani *et al.*, 2014). Some studies specifically highlight gaps that exist in national screening programmes for cancers such as breast, cervical and colorectal ( Satgé *et al.*, 2023; Sullivan *et al.*, 2003; Swaine *et al.*, 2014). Evidence in the intellectual disability population points towards factors such as limited screening education, distrust in healthcare providers, and challenges in providing consent, leading to limited uptake of available screening programmes and there appear to be misassumptions on women with intellectual disability not requiring cervical screening ( Power *et al.*, 2024; Swaine *et al.*, 2014; Weise *et al.*, 2024; Xu *et al.*, 2017). While there are many contributing factors to these inequalities, changes in individuals' health status may go unrecognised for significantly longer because of their intellectual disability ( Kiani *et al.*, 2014). In some cases, symptoms suggestive of cancer and cancer itself go undetected, and cancers are identified at a late stage or as a cause of death in individuals with intellectual disability ( Heslop *et al.*, 2022; Mahar *et al.*, 2024). Communicating symptoms of cancer may be difficult for individuals with an intellectual disability, and those around them may not notice signs of illness, both reasons which may contribute to a late-stage cancer diagnosis ( Satgé *et al.*, 2014). Ultimately this phenomenon, known as diagnostic overshadowing, may contribute to a decreased quality of life and the unnecessary progression of cancer, resulting in premature mortality ( Mason & Scior, 2004; McMahon *et al.*, 2024).

Although cancer is being diagnosed at more advanced stages in adults with intellectual disability, the evidence does not show that diagnosis occurs later in life ( Mahar *et al.*, 2024; Satgé *et al.*, 2014). In a study conducted by Satgé and colleagues, the age at which individuals with intellectual disability were being diagnosed with cancer was younger than the recommended screening age for the general population with Heslop *et al*. (2022) and Mahar *et al.* (2024) reporting similar findings in England and Canada respectively. This may be an indication that adults with intellectual disability are developing cancer earlier in adulthood, and screening protocols may need to be tailored to this. Recent evidence has shown that individuals with intellectual disability were 1.6 times more likely to be diagnosed with Stage IV breast cancer and 1.44 times more likely to be diagnosed with Stage IV colorectal cancer ( Mahar *et al.*, 2024). While the reasons why they are developing late-stage cancer at earlier ages have not yet been established, studies suggest that lowering screening ages for people with intellectual disability could be beneficial for earlier diagnosis. The prognosis for cancer patients is dependent on the cancer stage at diagnosis, so a later diagnosis in patients with intellectual disability could be a key factor accounting for differences in mortality rates.

There is limited research that focuses on treatment options for individuals with intellectual disability diagnosed with cancer. Much of the published evidence are case studies based on one individual's cancer experience with no synthesis of the published evidence available ( Brown, 2011; Enomoto *et al.*, 2015). In terms of diagnostic tools, while individuals with intellectual disability may utilise the same tools as the general population, tools such as mammography, Papanicolaou tests (pap smears), and testicular examinations, remain under-utilised by the intellectual disability population ( Satge *et al.*, 2023). The existing gaps in cancer diagnosis may be from patients not receiving regular screening ( Chan *et al.*, 2022; Sullivan *et al.*, 2003).

Although the exploration of treatments in case studies are specific to individual patients, these treatments may set a precedent that tailored treatment plans are required for the specific needs of this vulnerable population ( Brown, 2011; Delany *et al.*, 2023; Satgé *et al.*, 2014; Sleijfer *et al.*, 1996). Consequently, the aim of this literature review is to explore and synthesise information relating to cancer diagnostic approaches and treatment options for adults with intellectual disability and examine the barriers that prevent adults with intellectual disability from accessing both diagnostic tools and treatments.

## Methods

A literature review guided by the PRISMA Extension for Scoping Review checklist was undertaken to understand the experiences of adults with intellectual disability undergoing cancer diagnostic tests and treatments. Literature reviews provide a broad, thorough critique and examination of all the evidence available on the topic and facilitate a descriptive analysis and meaningful synthesis of the current available research ( Colquhoun *et al.*, 2014).

### 1. Research question

The PICOS (Population, Intervention, Comparison, Outcome, Study Type) framework was used to frame the research question and search concepts for this review. The research question to be addressed is:

*‘What are the barriers or enablers to accessing cancer diagnostics and treatment for adults with intellectual disability?’*

This will be achieved through two objectives:

• Explore and synthesise information relating to cancer diagnostic approaches and treatment options for adults with intellectual disability
• Examine barriers and enablers to accessing cancer diagnostic tools and treatments for adults with intellectual disability.

### 2. Eligibility criteria

The eligibility criteria are summarised in Table 1.

**Table 1.** Literature review eligibility criteria.

	Include	Exclude
**Population**	- Adults with all levels of intellectual disability - Down syndrome and other syndromes (if intellectual disability presence confirmed)	- Children, teenagers under 18 years of age - Adults without intellectual disability
**Intervention/Exposure**	- Any diagnosis and treatment of cancer	- Studies with no specified focus on diagnosis or treatment of cancer
**Comparison**	- Individuals living in residential, community group homes, with family or independently	- None. All living circumstances included.
**Outcome**	- Cancer (any type)	- Studies reporting outcomes not related to cancer
**Study Characteristics**	- Observational (retrospective and observational) - Qualitative - Cohort studies - Randomised control trials	- Reviews - Editorials or opinion pieces - Book chapters
**Language**	- English	- All non-English languages

### 3. Search strategy

Five electronic databases were systematically searched: Cinahl Ultimate, Medline, PsycINFO, PubMed, and Web of Science. To provide the most accurate search, search functionality and keywords were used and adjusted accordingly for each database. Keywords included “intellectual disability”, “treatment”, “diagnosis”, and “cancer”. Boolean operators were employed, including “and”, “or”, along with “not”. Table 2 shows an example of the MEDLINE search string. Finally, citations were searched in relevant reviews about cancer treatments and screening for adults with an intellectual disability. All articles were extracted to Covidence which was used as the screening tool.

**Table 2.** An example of the MEDLINE search string.

Concept	Index	Keywords
**Concept 1:** Cancer	(MH “Cancer”)	(Cancer OR oncology OR neoplasm OR malignant OR malig*)
**Concept 2:** Intellectual disability or learning disability	(MH “Intellectual Disability+”) OR (MH “Learning Disabilities+”)	((intellectual AND disabilit* OR ‘mental retardation’/exp OR ‘mental retardation’ OR (mental AND (‘retardation’/exp OR retardation)) OR ‘learning’/ exp OR learning) AND disabilit* OR developmental) AND disabilit* OR ‘learning disabilities’/exp OR ‘learning disabilities’ OR ((‘learning’/exp OR learning) AND disabilities)
**Concept 3:** Diagnosis	(MH “Diagnosis”)	(diagnos* OR screen* OR assess* OR evaluation OR detect* OR identif*)
**Concept 4:** Treatment	(MH “Therapeutics”)	(treat* OR therap* OR intervention OR management OR care OR “therapeutic approach” OR “clinical management”)

### 4. Screening process

**Figure 1.**
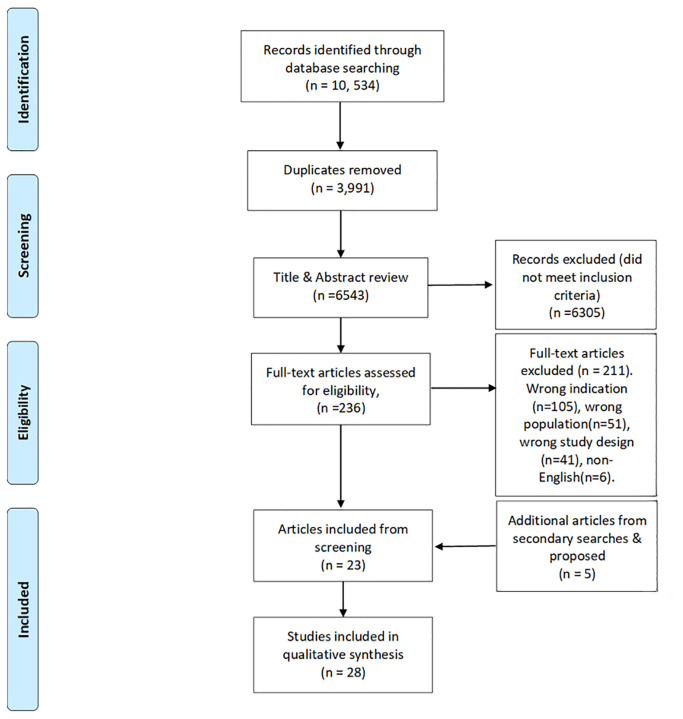
PRISMA diagram.

Figure 1 summarises the screening process in a PRISMA diagram. Two assessors [KS, MD] reviewed each of the articles. A third [LL or MMcM] adjudicated in cases of dispute. The initial search resulted in 10,534 publications with 3,991 duplicates removed. A title and abstract screening of 6,543 articles was completed. Then, 236 articles proceeded to full-text review. Finally, 23 articles were extracted, along with three articles from citation searching and two additional proposed articles, resulting in a total of 28 articles for inclusion in the literature review.

### 5. Data extraction

Data was gathered from studies conducted worldwide from 1996 to 2024. The following information was extracted from each article: author, year, country, study aim, study design, sample size, age, gender, results and theme, see Table 2. All information was not available for each article.

### 6. Thematic analysis

A qualitative synthesis and thematic analysis was completed on the extracted articles using the Braun and Clark Six Step process, see Table 3 ( Braun & Clarke, 2006). Initially the researchers familiarised themselves with each article’s content by reading multiple times and making notes on each, to fully immerse themselves in the data. As initial patterns emerged, colour-coding was used to group similar codes together. Broad themes emerged as a result of this grouping. These broad themes were then refined further, analysing the codes that were contained in each theme until four broad themes remained. Some articles were grouped into multiple themes as they covered multiple topics.

**Table 3.** Six step thematic analysis process.

Step number	Process	Explanation
**1**	Data familiarisation	Complete data immersion
**2**	Generate initial codes	Topics, patterns of data
**3**	Search for themes	Broader theme identification
**4**	Review of themes	Theme refinement
**5**	Define and name themes	Categorise. Include sub-themes if required
**6**	Produce report	Complete write-up

( Braun & Clarke, 2006)

## Results

A total of 28 articles were included in the final review. After completing the Braun and Clark thematic analysis, the four overriding themes emerged i.e. Prevention, Education, Adaptation and Ethical practice. Some overlap was observed in articles that covered multiple themes ( Armin *et al.*, 2022; Brown, 2011; Flynn *et al.*, 2016; Sleijfer *et al.*, 1996; Sullivan *et al.*, 2003). The number of people with intellectual disability represented in this review was 119,909. Studies were representative of a worldwide population; USA (n=7), UK (n=6), Canada (n=5), France, Australia and Japan (n=2), and Sweden, Denmark, Taiwan and The Netherlands (n=1). Overall, 16 articles investigated preventative measures of cancer screening, seven articles discussed education on cancer and screening for people with intellectual disability, seven articles reviewed cancer treatment options for patients with intellectual disability, and three articles discussed patient autonomy, which was covered by ethical practices, see Table 4a and Table 4 in extended data. A summary of each theme’s results is provided. Screening was addressed in prevention and education. Treatments were covered by adaptation and ethical practice.

**Table 4a.** Article extraction summary.

Author, year	Country	Aim	Design	Age	% Male	No of participants	Results	Theme
Armin *et al.*, 2022	USA	Identify barriers to cancer screenings in native American patients with intellectual disability	Semi-structured interviews	18–44	7%	n=48	Individual, interpersonal, and community/institutional barriers to screenings due to social inequities. They were then utilised in creating a cancer screening education program.	Prevention, Education
Bates and Triantafyllopoulou, 2019	UK	Determine barriers and mental capacity impact on breast cancer screenings for women with intellectual disabilities	Cross-sectional survey	50–70	0%	n=131	Barriers to screening include poor mobility and behavioural difficulties. Also, women who lacked mental capacity were less likely to engage in the screenings.	Prevention
Brown *et al.*, 2016	Canada	To compare cervical cancer screening rates in women with and without intellectual disability who had a pregnancy	Retrospective cohort	29–64	0%	n=5,033 (with intellectual disability), n=527,437 (without intellectual disability)	Women with intellectual disability were less likely than women without intellectual disability to be screened, after controlling for health and social factors.	Prevention
Cobigo *et al.*, 2013	Canada	To estimate rates of cervical & breast cancer screening among eligible women with intellectual disability & compare to women without intellectual disability; To examine if any observed differences between the groups persist after certain factors are accounted for	Cross-sectional using health administrative data and registries	20–69	0%	n=17,777 with intellectual disability, n=1,440,962 without intellectual disability	Women with intellectual disability are almost twice less likely to be screened for cervical cancer and 1.5 less likely to receive mammograms than women without intellectual disability.	Prevention
Flynn *et al.*, 2016	UK	Explore the experiences of people who have an intellectual disability and their diagnoses and treatment of cancer	Interviews conducted and analysed using Grounded theory	34–77	N/A	Patients with Intellectual disability & cancer n=6, Family & healthcare professionals n=12	People with intellectual disability were often excluded from decisions in their cancer care and treatment. However, when they were included, there were positive outcomes with meaningful engagement of their care. Therefore, more methods put into play in aiding patients with intellectual disability to understand their diagnoses	Prevention, Ethical practice
Heslop *et al.*, 2022	England	Expand the current understanding of cancer and screenings in adults with intellectual disabilities	Population based study	20+	n/a	n=1096	In individuals with intellectual disabilities who were deceased, cancers were presented in emergency situations, 45% being stage IV cancers at diagnosis. Of these cancer related deaths, 36% were digestive system cancers and 48% of these were colon cancers. 43% of the patients who died of colon cancer were below the screening age recommended for the general population.	Prevention
Horsbøl *et al.*, 2023	Denmark	Compare breast cancer screening	Cohort study	50–69	0%	Patients with intellectual disability n=5595, Patients without intellectual disability n=49,423	25% of patients with intellectual disabilities were fully screened compared to 62% of patients who did not have intellectual disabilities. Additionally, 45% of patients with intellectual disabilities had never been screened compared to 13% in the population that does not have intellectual disability. As patients' severity of intellectual disability increased their likelihood of screening decreased.	Prevention
Kiani *et al.*, 2014	UK	Case of an individual with a profound Intellectual disability who developed a chest infection, and recurrent infections after that and was then diagnosed with cancer after death in the patients’ autopsy	Case study	56	100%	n=1	The patient's cancer symptoms had been attributed to his chest infections, and whether an early diagnosis could have altered the patient's outcome, the health care team should have further investigated these symptoms.	Prevention
Lai *et al.*, 2014	Taiwan	Investigate the rate of uptake of mammography and pap smears in patients with intellectual disability. Then, determine external factors that may influence utilising these cancer screening methods.	Population based study	50–69	0%	n=4370	The mammography utilisation rate for women with intellectual disability was 4.32% compared to the general population of 12%. There were external factors also discovered that could lead to increased or decreased utilisation including marriage, education, and other preventive health use.	Prevention
Mahar *et al.*, 2024	Canada	Determine the incidence and disparities between people with intellectual disabilities and their delayed diagnoses for breast, colorectal, and lung cancers	Multiple population-based cross-sectional studies	49–80	Breast cancer- 0% Colorectal cancer- 54% Lung cancer- 50.3%	Patients with intellectual disability & breast cancer n=83, Patients with breast cancer, n=10,610, Patients with intellectual disability & colorectal cancer n=84, Patients with colorectal cancer n=10,283, Patients with intellectual disability & lung cancer n=67, Patients with lung cancer n=11,400	Patients with intellectual disabilities were 1.6 times more likely to be diagnosed with breast cancer and 1.44 times more likely to be diagnosed with colorectal cancer at stage IV compared to patients without intellectual disability due to low screening rates and delayed diagnosis.	Prevention
Ouellette-Kuntz *et al.*, 2015b	Canada	Examine the participation of patients with intellectual disabilities in colorectal cancer screenings	Routine-data-based study	50–64	53.9% with Intellectual disability, 49.4% without	Patients with Intellectual disability n=15,791, Patients without n=791,792	In each age range for participants, the percentage of males with intellectual disabilities was at least 10.7% lower than their counterparts without intellectual disabilities.	Prevention
Ouellette-Kuntz *et al.*, 2015a	Canada	Compare the utilisation of secondary screenings and prevention in adults with and without intellectual disabilities	Retrospective cohort study	18–64	19.9% with Intellectual disability, 20.5% without	Patients with intellectual disabilities n= 66,484, Patients without intellectual disabilities n=2,760,670	26.4% of adults without intellectual and developmental disabilities had a regular health examination over the two-year period in comparison to the 22% of individuals with intellectual disabilities. The study found that adults with intellectual and developmental disabilities were less likely to undergo recommended screenings for breast, colorectal and cervical cancer.	Prevention
Satgé *et al.*, 2023	France	Examine the colorectal screening rates for patients with intellectual disabilities and what stage these patients are being diagnosed with colorectal cancer. Then, compare these rates with the general population and determine any disparities if present	Registry review	32–85	35.70%	n=14 patients	64.3% of the time patients with intellectual disability are diagnosed with colorectal cancer at stage IV compared to the general population at 26%. Out of these patients 10 died soon after their diagnosis due to advanced tumour stages not allowing for treatments. It was discovered that people with intellectual disabilities do not participate in as many faecal occult blood tests which is a common screening for colorectal cancer and may be the reason they are receiving delayed diagnosis. Therefore, it is important to increase the number of screenings for this population.	Prevention
Satgé *et al.*, 2014	France	Compares age of diagnosis, tumour size, Scarff-Bloom-Richardson grading, TTMN classification and AJCC stage in women with and without Intellectual disability	Retrospective study	40–55	0%	484 total, n=11 with intellectual disabilities	In women with intellectual disabilities, breast cancer was diagnosed at a mean age of 55.64 years while the mean age in women without intellectual disabilities was 62.35. The tumour size in women with intellectual disabilities was found to have a range of 1.5–8 cm which is greater than the control group.	Prevention
Sullivan *et al.*, 2003	Australia	Compare the incidence of breast cancer and the uptake of mammography screening services by women with intellectual disabilities	Audit based on Disability Services Commission database	25+	0%	n=2,370	34.7% of patients with intellectual disabilities utilised breast cancer screening methods such as mammograms compared to 54.6% in the general population. This proves the underutilisation of these services in this population.	Prevention, Education
Tuffrey-Wijne *et al.*, 2010)	UK	Explore how much people with intellectual disabilities are told and understand about their diagnosis of cancer	Ethnographic study	36–66	53%	n=13	11 out of 13 patients were told they had cancer, but most did not understand their diagnosis or prognosis and were kept apart from the decision-making process of their care and treatment. It was also determined that the worse the persons' intellectual disability was the lessened their probability of being told about their diagnosis and had a lower sense of understanding.	Prevention, Ethical practice
Armin *et al.*, 2023	USA	An education programme for native American women with intellectual disability called "My Health, My Choice" in hopes of increasing screening uptake for this population	Pre- and post-survey	18–44	0%	n=12	The patients were able to undergo surgeries that are not normally applicable for this population. This shows that these types of treatments that normally are not options for this population can be accessible with minor modifications and lead to improved health outcomes	Education
Greenwood *et al.*, 2014	USA	Evaluate acceptability, demand and limited efficacy of a health education DVD about mammography for women with Intellectual disability	Feasibility study	37–82	0%	n=27	The DVD was proven successful as the patients demonstrated increased knowledge of mammography screenings. All the participants except one rated highly the statement that they learned a lot from the DVD, which had a rating overall of 4.4 and all the participants except four requested to keep the DVD.	Education
Swaine *et al.*, 2014	USA	Evaluate if the version Women Be Healthy 2 is effective in increasing the knowledge of women with Intellectual disability regarding breast & cervical screening	Randomised control trial	36–37	0%	n=198	Women with intellectual disabilities who underwent the Women Be Healthy 2 programme demonstrated an increased understanding of breast and cervical screenings in comparison to women with intellectual disabilities who did not undergo the programme.	Education
Wang *et al.*, 2015	USA	To determine the validity of the Mammography Preparedness Measure	Pilot testing of a measurement instrument, followed by assessing the test-retest reliability of the Mammography Preparedness Measure	37+	0%	n=48	The Mammography Preparedness Measurement was found to be a valid instrument for assessing the preparedness of women with intellectual disabilities to receive a mammogram. The tool was found to have a test-retest per cent agreement of 84%.	Education
Wilson *et al.*, 2018	UK	To assess and compare the impacts that a leaflet informational packet and an educational programme utilising videos have on testicular health	Participatory randomised parallel study of two educational interventions	16–34	100%	n=192	One week after the intervention the educational programme group scored significantly higher in an assessment of their knowledge and skills of testicular health. However, in from week 1 to 6 months both the leaflet and educational programme groups demonstrated this increase in knowledge and skills. Additionally, there was a significant increase in self-efficacy in both groups where individuals felt confident enough to perform self-testicular exams.	Education
Brown, 2011	USA	Patients with intellectual disability receiving allogeneic transplant and other aggressive cancer treatments without understanding their options	Case-study audit	n/a	100%	n=1	The patient passed away within a year of the transplant and follow up treatment due to pneumonia and underwent visible suffering throughout	Adaptation, Ethical practice
Delany *et al.*, 2023	Australia	Modified chemotherapy and communication methods to tailor patient needs & Intellectual disability	Case-study audit	33	100%	n=1	Patient made a complete recovery after chemotherapy with the help of collaboration and communication between clinical teams, family and carers throughout the treatment. Paper argues this should be the new standard for this population.	Adaptation,
Enomoto *et al.*, 2015	Japan	Case of oral cancer and the three rounds of modified and specialised chemotherapy they underwent using infusions to the femoral artery	Case-study audit	32	100%	n=1	The patient made a great recovery and has had no signs of recurrence. The paper explains further how much more successful treatments can be for this population with modifications to meet their needs.	Adaptation
Gandhidasan *et al.*, 2020	USA	Outpatient anaesthesia facilitating delivery of stereotactic body radiation therapy in patients with severe cognitive impairments and inoperable lung cancer.	Retrospective review	44–78	38%	n=7	Outpatient anaesthesia was tolerated by the patients and the stereotactic body radiation therapy was able to be completed in patients with cognitive impairments. There was one stereotactic body radiation related toxicity that resulted in grade 5, however there were no other toxicities above grade three found.	Adaptation
Ishimaru *et al.*, 2022	Japan	Investigate Tumour Screening, Incidence, and Treatment for Patients Intellectual Disabilities	Chart review	27–67	50%	n=12	The patients were able to undergo surgeries that are not normally applicable for this population. This shows that these types of treatments that normally are not options for this population can be accessible with minor modifications and lead to improved health outcomes	Adaptation
Segerlantz *et al.*, 2019	Sweden	Comparison of prescription pain medications in cancer patients with intellectual disabilities, and cancer patients in the general population	National registry cohort study	55 years +	n/a	n=555 patients with Intellectual disability, n=877 cancer patients from the general population	Cancer patients with intellectual disabilities are less likely to be prescribed COX inhibitors, and weak opioids, but more likely to be prescribed paracetamol, and antidepressants than cancer patients in the general population.	Adaptation, Ethical practice
Sleijfer *et al.*, 1996	Netherlands	Report on a patient who has down syndrome and a disseminated seminomatous tumour of the testis and what their treatment entailed	Case study	30	100%	n=1	Cisplatin-based regimens of chemotherapy are difficult for patients with intellectual disability to undergo though this patient had remarkable success with a carboplatin-containing chemotherapy, since it is available to be administered on an out-patient basis making it an improved alternative to the former.	Adaptation, Ethical practice

### (a) Prevention

The importance of cancer screening as a preventative measure for people with intellectual disability due to noted higher risks of developing cancer and having difficulties with explaining their pain and symptoms, was discussed in 14 articles. Armin *et al.* (2022) found that social inequalities contributed as barriers to screening for adults with an intellectual disability while intellectual disability severity level was negatively associated with screening levels ( Horsbøl *et al.*, 2023). Additionally, this population were less likely to receive cancer screening in comparison to the general population due to unique barriers including living conditions, and distress and anxieties they experience may make them less likely to engage with screening ( Tuffrey-Wijne *et al.*, 2010). For example, Canadian women with intellectual disability are less likely to receive cervical screening than women without intellectual disability, in some cases almost twice less likely, even if they were confirmed to be sexually active, and 1.5 times less likely to receive mammograms ( Brown *et al.*, 2016; Cobigo, 2013). Similarly, a Taiwanese study reported that the breast cancer screening and cervical screening rates for women with intellectual disability was almost three times and six times less than women in the general population ( Yen *et al.*, 2014). In addition, women with intellectual disability are five times more likely to never be screened for cancer than the general population and a lower rate of mammograms is observed in women with intellectual disability ( Ishimaru *et al.*, 2022; Lai *et al.*, 2014). Similarly, men with intellectual disability had lower rates of cancer screening. A Canadian routine data-based study on colorectal cancer (n=807,583) reported that 18.3% of intellectual disability participants received a faecal occult blood test in the previous two years, while 32% were up to date with their colorectal screening, compared to the general population at 26.4% for screening in the last two years and 47.2% for being up to date with their screening ( Ouellette-Kuntz *et al.*, 2015a).

Furthermore, mobility, behavioural issues and a lack of mental capacity emerged as barriers for women with intellectual disability receiving cancer screening ( Bates & Triantafyllopoulou, 2019). Lower levels of cancer screening resulted in increased numbers of late-stage cancer diagnoses with higher levels of poor health outcomes ( Heslop *et al.*, 2022; Satgé *et al.*, 2014). A concern is that people with intellectual disability have presented with cancer at ages below those recommended for screening commencement for the general population ( Heslop *et al.*, 2022; Mahar *et al.*, 2024; Satgé *et al.*, 2023).

### (b) Education

Education about cancer screening and treatments was explored in seven articles. Various screening educational tools were used, including the ‘Mammography Preparedness Measure’ which assessed women’s readiness for Mammograms and a testicular cancer education programme which resulted in improved self-efficacy among men with intellectual disability ( Wang *et al.*, 2015; Wilson *et al.*, 2018). An American ‘My Health My Choice’ program demonstrated that education resulted in treatment options being offered that would otherwise not be presented ( Armin *et al.*, 2023). The American ‘Women Be Healthy 2’ program and DVDs as educational tools were positively received by adults with an intellectual disability and demonstrated an increase in knowledge of screening ( Greenwood *et al.*, 2014; Swaine *et al.*, 2014).

### (c) Adaptation

The modification of treatments to make them more amenable to patients with intellectual disability was the third theme that emerged in this review. Specific cancer treatments for patients with intellectual disability were detailed in seven articles. Adapted chemotherapy was the focus of three articles, where the successful adjustment of treatments to meet the specific needs of the patients resulted in positive outcomes ( Delany *et al.*, 2023; Enomoto *et al.*, 2015; Sleijfer *et al.*, 1996). An article with a focus on radiation therapy showed that anaesthesia could be used to improve treatment tolerance ( Gandhidasan *et al.*, 2020). A standard approach to cancer surgery for those with and without intellectual disability showed that similar results were achievable ( Ishimaru *et al.*, 2022). A study which investigated pain management for cancer patients found that people with an intellectual disability were less likely to receive opioids, compared to their peers in the general population ( Segerlantz *et al.*, 2019). The authors proposed that this lack of treatment was due to communication challenges rather than the absence of pain. Concerningly, a case study on a stem transplant for a patient with intellectual disability who lacked the understanding of the procedure, resulted in premature death and unwarranted suffering ( Brown, 2011). Some overlap was observed within studies where other treatments were also incorporated.

### (d) Ethical practice

Ethical practice was the final broad theme, which included autonomy, where patients with an intellectual disability were able to decide on their own treatment options e. Patient autonomy explored the unique role of care partners and their roles in supporting this population in health matters. More positive outcomes and meaningful engagement were seen with the proactive engagement of the patient in their own care while not updating the patient on their condition resulted in needless distress and an early death ( Brown, 2011; Flynn *et al.*, 2016). Studies also discussed the principle of “truth-telling” which is when medical providers and care partners determine how much, if at all they decide to explain to the patient about their diagnoses ( Bernal & Tuffrey-Wijne, 2008). Patient autonomy highlighted the ethical debates that surround consent and patients with an intellectual disability ( Brown, 2011; Bernal & Tuffrey-Wijne, 2008; Flynn *et al.*, 2016). Lastly, an equality in the distribution of prescription pain medication was observed, where older adults with cancer and intellectual disability were less likely to be prescribed pain medications then their general population peers resulting in the under-management of pain and a reduction in quality of life ( Segerlantz *et al.*, 2019).

## Discussion

This literature review examines the cancer diagnostic approaches and treatments available for adults with intellectual disability and any associated barriers or enablers. Results identified the importance of tailored education and application to cancer screening, diagnosis and treatment, and more involvement of adults with intellectual disability in decisions about their own care, on positive impacts on their cancer outcomes. Unique barriers to screening access were identified. Overall, the high number of individual case studies and the low numbers of people with intellectual disability in cancer studies is indicative of an under-researched area.

Although the average lifespan of people with intellectual disability has increased, as theoretically has the risk for cancer developing, they are still not receiving equitable screening to the general population ( Flynn *et al.*, 2016; Lai *et al.*, 2014; Mahar *et al.*, 2024; Satgé *et al.*, 2014; Wilkinson *et al.*, 2011).

By utilising secondary prevention, to diagnose cancer while it is still asymptomatic, cancer may be diagnosed earlier while it is still possible to have positive health outcomes ( Ouellette‐Kuntz *et al.*, 2015a). Nevertheless, individuals with intellectual disability may be unable to recognise signs and symptoms suggestive of cancer or be able to communicate their symptoms to a caregiver, and the caregiver may be unable to recognise symptoms which could make them more easily overlooked unless regular checks occur ( Ouellette-Kuntz *et al.*, 2015a).

A key risk factor for cancer is ageing, and screening for cancer detection is recommended at specific ages. Historically, the intellectual disability population had shorter lifespans, and many would not reach the age when preventative measures commenced and therefore were excluded from standard screening ( Flynn *et al.*, 2016; Lai *et al.*, 2014; Mahar *et al.*, 2024; Satgé *et al.*, 2014; Wilkinson *et al.*, 2011). However, people with intellectual disability may not receive necessary cancer screening due to a unique set of barriers ( Bates & Triantafyllopoulou, 2019; Delany *et al.*, 2023; Ouellette-Kuntz *et al.*, 2015b).

Where people resided was identified as a barrier to screening access ( Sullivan *et al.*, 2003; Xu *et al.*, 2017). People who lived in institutionalised care facilities had less screening levels, perhaps due to higher levels of intellectual disability and less resources at the facility ( Armin *et al.*, 2022; Sullivan *et al.*, 2003). Additionally, if a patient lives alone in a rural environment, a geographical influence is seen where transportation to screening may be difficult, often leading to less screening attendance ( Yen *et al.*, 2014). Conversely, one study showed a protective factor for those who resided in a group home or medical facility, where they were more likely to receive cancer screening due to established policies and procedures ( Xu *et al.*, 2017).

Elevated rates of late diagnosis are reported for people with intellectual disability compared to the general population. Studies indicate that patients with intellectual disability were more likely to detect breast, colorectal, and lung cancer at stages III and IV rather than stages I or II ( Mahar *et al.*, 2024; Satgeé *et al.*, 2023). These late-stage diagnoses of cancers meant that only limited treatments were available, and a high mortality rate occurred. Furthermore, people with intellectual disability have an onset of tumours at earlier ages than that of the general population, so normal screening timelines are inadequate for a timely diagnosis ( Heslop *et al.*, 2022; Mahar *et al.*, 2024).

Cognitive deficits associated with intellectual disability may make understanding cancer screening procedures or communicating with relevant specialists more difficult, which can heighten stress and anxiety surrounding procedures, resulting in their cancellation ( Bates & Triantafyllopoulou, 2019; Horsbøl *et al.*, 2023; Wilkinson *et al.*, 2011). A need for education on cancer screening was identified as critically important, to enable a person with intellectual disability to engage with screening. Many individuals with intellectual disability are not educated about the need for cancer screening or the processes involved, and often do not feel prepared to attend screening, which reduces uptake ( Wilkinson *et al.*, 2011). A lack of education and preparation has contributed to large gaps in cancer screening ( Ishimaru *et al.*, 2022). Females with intellectual disability expressed feelings of confusion, and felt they could not communicate their emotions with their providers and that their doctors did not understand the extent of their confusion ( Wilkinson *et al.*, 2011). Furthermore, patients felt they were misinformed or given inadequate information about screening procedures resulting in inadequate preparation. Results suggested that health professionals should take the time to explain to individuals with intellectual disability the specific details of procedures and provide additional support options such as videos for clarity ( Sullivan *et al.*, 2003; Wilkinson *et al.*, 2011).

A link between higher levels of education and higher levels of screening engagement has been observed which indicates a focus on education and reasonable adjustments for people with intellectual disability is necessary to increase cancer knowledge, confidence and screening participation rates ( Armin *et al.*, 2022; Greenwood *et al.*, 2014; Swaine *et al.*, 2014; Wang *et al.*, 2015; Wilson *et al.*, 2018). While education is an important foundation for supporting patients with intellectual disability, it is crucial that this is provided in conjunction with adaptations for cancer treatments ( Armin *et al.*, 2023). Case studies have demonstrated that modified chemotherapy regimens are feasible for the successful treatment of cancer for patients with intellectual disability and concluded that following evidence-based practice is not always the best route of cancer care for individuals with intellectual disability, and that health care providers need to take time to make the necessary care adjustments ( Delany *et al.*, 2023; Enomoto *et al.*, 2015). These case studies, while limited in numbers, demonstrated that individuals with intellectual disability should be considered for chemotherapy treatments, and providers should tailor treatment and care to their specific needs.

Radiation and surgery are often an integral part of cancer treatment. Both have been successfully adapted and employed in patients with intellectual disability, where individualised care based on the unique needs of the patients with intellectual disability was considered ( Gandhidasan *et al.*, 2020; Ishimaru *et al.*, 2022). While there are ethical debates about men with testicular cancer undergoing radical inguinal orchidectomy for both diagnosis and treatment, the cure rate can be increased by finding an appropriate balance of therapy ( Hafeez *et al.*, 2015).

Indeed, ethical principles play a large role in the type of cancer treatment that individuals with intellectual disability receive. A case study on a stem cell transplant largely focussed on treatment ethics, although this known risky therapy was not proven to be successful because of the complications the patient experienced, independent of the presence of an intellectual disability ( Brown, 2011). Similarly, studies explored debates regarding pain management and prescription drugs being used in the treatment of individuals with intellectual disability ( Axmon *et al.*, 2018; El-Tallawy *et al.*, 2023; Millard & de Knegt, 2019; Segerlantz *et al.*, 2019). Older adults with cancer and intellectual disability were less likely to receive prescription pain medications then their counterparts resulting in the under-management of pain and a reduction in quality of life ( Segerlantz *et al.*, 2019).

The ethical debate of treating cancer patients with intellectual disability continues into the theme of autonomy. Having an intellectual disability may inhibit an individual's understanding of a plethora of topics which may further alter how decisions are made ( Hafeez *et al.*, 2015). Nonetheless, the intellectual disability community has a long history of debating how to handle decision-making and notifying patients with intellectual disability of different diagnoses, especially when it comes to cancer, complicated by individual country legal frameworks and capacity ( Delany *et al.*, 2023; Kiani *et al.*, 2014; Tuffrey-Wijne *et al.*, 2010). Often people with intellectual disability have difficulties being independent and may have carers who have influence over their medical, educational, and financial decisions ( Kiani *et al.*, 2014). A lack of truth-telling stems from wanting to protect the patient from excess anxiety and distress, though it may limit the patient’s autonomy. However, there is a lack of evidence about distress after cancer diagnosis in patients with intellectual disability, implying that these patients should be entitled to honesty when it comes to their care and making medical decisions ( Bernal & Tuffrey-Wijne, 2008; Flynn *et al.*, 2016). Furthermore, medical professionals participated in non- “truth-telling” due to feelings of discomfort when working with this population, and they conferred solely with care partners about the patient's condition rather than with the patient themselves ( Flynn *et al.*, 2016; Tuffrey-Wijne *et al.*, 2010). Studies also found evidence where carers have asked for the professionals to discuss diagnosis with the patients and the professionals refused, most likely due to a lack of confidence or understanding of intellectual disability. Brown explains an ethical dilemma where a patient who was unable to consent for themselves underwent aggressive treatment, in line with best practice, despite doubts raised by nursing staff ( Brown, 2011). The patient passed away from pneumonia within a year of treatment, which brings into question the overall suitability of the treatment. Informed consent is a demonstrated successful consent method for patients with intellectual disability and should be used as part of a standard protocol. These findings may serve as a guide for healthcare professionals in providing diagnostic and treatment options, educating their patients, and communicating with patients with intellectual disability about their illness. Cancer patients with intellectual disability need to have their voices included in the research.

## Conclusion

This literature review highlights substantial differences in treatment and diagnosis needs in cancer patients with intellectual disability and their general population peers. It provides evidence of the lack of research in the area where case studies dominate and the urgent need to cater for this underserved, underrepresented population. Unique barriers to screening including living conditions, age, life span and communication difficulties, contributed to later diagnosis and worse cancer outcomes.

This review demonstrates that people with intellectual disability should be provided with more education on cancer screening and treatment options and should have access to screening at earlier ages. Finally, this literature review showed that in order to preserve ethical principles, people with intellectual disability should be provided with more tailored treatment options, their autonomy should be given due consideration and truth-telling should be practiced, which could improve their cancer diagnosis and treatment outcomes.

## Limitations

This literature review has several limitations including an over reliance on case studies with the heterogeneity of study designs making synthesis more challenging. Given the extensive body of literature, it is possible that some relevant studies were not included. Additionally, cancer diagnosis and treatment may have a geographical residential influence which may impact study findings. Equally, the subjective nature of thematic analysis may affect the interpretation and applicability of findings. Lastly, aged results are reported due to a dearth of recent evidence.

## Data Availability

No data were associated with this article Harvard Dataverse- **Replication Data for: Table 4: Article extraction summary information** https://doi.org/10.7910/DVN/VD23QF ( Lynch, 2025) Data available under CC0 1.0 licence
