# Supplementary material for: A literature review of cancer diagnostic tests and treatments in adults with intellectual disability
Source: HRB Open Res. 2026 Apr 15;8:66. Originally published 2025 Jun 13. [Version 3] doi: 10.12688/hrbopenres.14164.3 (PMC13000398; doi:10.12688/hrbopenres.14164.3)
Supplement: Peer review history

# Reviewer report: Reviewer response for version 3

**DOI:** 10.21956/hrbopenres.15890.r54650

**Published:** 2026-04-23

**Authors:** Kumaresan Cithambaram

**Affiliations:** 1 Technological University of the Shannon, Athlone, Ireland

The authors have addressed all comments and queries satisfactorily. I recommend approval for Indexing.

Are the rationale for, and objectives of, the Systematic Review clearly stated?

Yes

Is the statistical analysis and its interpretation appropriate?

Not applicable

Are sufficient details of the methods and analysis provided to allow replication by others?

Yes

Are the conclusions drawn adequately supported by the results presented in the review?

Yes

Reviewer Expertise:

Palliative care, intellectual disability, end-of-life care, migrant health, systematic review.

I confirm that I have read this submission and believe that I have an appropriate level of expertise to confirm that it is of an acceptable scientific standard.

# Reviewer report: Reviewer response for version 2

**DOI:** 10.21956/hrbopenres.15784.r53851

**Published:** 2026-03-18

**Authors:** Kumaresan Cithambaram

**Affiliations:** 1 Technological University of the Shannon, Athlone, Ireland

Thank you for the opportunity to review this paper. The objectives and rationale of the review are clearly outlined, and the paper provides insight into the needs of, and support for, people with intellectual disabilities when they receive screening related to cancer diagnosis and treatment. The methodology is clearly described and follows the PRISMA Guidelines, and the conclusions are appropriately drawn from the review findings. However, several suggestions are offered below to strengthen the manuscript.

**Abstract:**

 Background:

 Consider revising “diagnostic tools” to “diagnostic procedures.” The review seems to address access to diagnostic processes rather than specific tools, so access to diagnostic procedures and treatments may be a clearer and more accurate description.

 Method:

 Consider replacing “analysis” with “synthesis,” as synthesis is generally the more appropriate term in the context of literature or systematic reviews, where findings from multiple studies are integrated rather than analysed as primary data.

 Results:

 There appears to be an inconsistency between the abstract and the findings section. The findings describe “ethical issues” as a theme, while the abstract refers to “autonomy.” The results section of the abstract should be revised to reflect the themes reported in the findings.

**Introduction**

 Check citation style: Loud & Murphy (2017) or ( Loud & Murphy, 2017)

 Paragraph 2

 The statement *“there appear to be misassumptions that women with intellectual disability do not require cervical screening”* requires further clarification. Please specify whose assumptions are being referred to (e.g., healthcare professionals, family members, or caregivers) and explain the basis of these assumptions. For example, are they related to beliefs that women with intellectual disability are not sexually active? This would benefit from further explanation and discussion.

 The description of diagnostic overshadowing appears inaccurate. Diagnostic overshadowing refers to healthcare professionals misattributing physical symptoms to a person’s intellectual disability rather than investigating possible medical causes. Please review and revise this explanation.

 This statement requires supporting evidence “While the reasons why they are developing late-stage cancer at earlier ages have not yet been established, studies suggest that lowering screening ages for people with intellectual disability could be beneficial for earlier diagnosis”

 This statement requires supporting evidence “The prognosis for cancer patients is dependent on the cancer stage at diagnosis, so a later diagnosis in patients with intellectual disability could be a key factor accounting for differences in mortality rates”

**Methods:**

**Eligibility Criteria**

 The acronym PICOS may not be appropriate for this review. PICOS is typically used to compare interventions and outcomes, whereas this review does not compare across different living environments, and the outcome does not measure cancer itself. Rather, the review aims to identify barriers and enablers to access to cancer diagnostic procedures and treatment.

 Consider using the PICoS framework instead:

 P – Population: Adults with intellectual disability

 I – Intervention: Cancer diagnostic procedures and treatment

 Co – Context: Barriers and enablers influencing access to cancer diagnostic procedures and treatment

 S – Study type

 Consider revising the framework to PICoS or another more appropriate acronym that reflects the focus of the review.

**Screening Procedure**

 PRISMA flow diagram: Please review the numbers reported at each stage, as there appear to be inconsistencies.

**Data extraction**

 Please include a discussion of how the data extraction table was developed, who conducted the data extraction, and how discrepancies between

 reviewers were resolved.

**Thematic Analysis**

 Consider replacing “analysis” with “thematic synthesis,” as this term is more appropriate for review methodology. Please also include a discussion on who conducted the synthesis and how codes and themes were developed and agreed.

**Results**

 Statement “see Table 4a and Table 4 in extended data” Table 4 repeated.

**Ethical Issues**

 Remove “e” at the end of the sentence “Ethical practice was the final broad theme, which included autonomy, where patients with an intellectual disability were able to decide on their own treatment options e”

 Consider using “individuals” or “adults with intellectual disability” rather than “patients,” where appropriate, to maintain person-centred language.

 Consider replacing “medical providers” with “healthcare professionals,” “medical professionals,” or “healthcare providers.”

**Discussion**

 The discussion section would benefit from being more focused. Please tighten this section to concentrate on the themes identified in the review and support these with relevant literature. Some parts of the discussion are quite broad and repeat points already presented in the introduction, for

 example: *“Although the average lifespan of people with intellectual disability has increased, and theoretically the risk of cancer has also increased, they are still not receiving screening at the same rate as the general population.”*

 A sentence begins with *“A link between higher levels of education…”*. Please clarify what is meant by “higher levels of education.” Does this refer to higher educational qualifications or to greater knowledge and understanding of cancer screening and information? This should be clarified.

 The sentence *“While education is an important foundation for supporting patients with intellectual disability, it is crucial that this is provided in conjunction with adaptations for cancer treatments”* requires clarification. Please specify who the education is intended for. Does this refer to education for individuals with intellectual disability, or training/education for healthcare professionals and caregivers supporting them?

 Consider including a section on the implications of the review, outlining how the findings contribute to practice, research, and policy.

 I suggest using the term “education” carefully throughout the paper to clarify that it refers to education and information related to cancer screening and treatment, rather than the educational level of adults with intellectual disability.

Are the rationale for, and objectives of, the Systematic Review clearly stated?

Yes

Is the statistical analysis and its interpretation appropriate?

Not applicable

Are sufficient details of the methods and analysis provided to allow replication by others?

Yes

Are the conclusions drawn adequately supported by the results presented in the review?

Yes

Reviewer Expertise:

Palliative care, intellectual disability, end-of-life care, migrant health, systematic review.

I confirm that I have read this submission and believe that I have an appropriate level of expertise to confirm that it is of an acceptable scientific standard, however I have significant reservations, as outlined above.

# Response

**Published:** 2026-03-30

**Authors:** Louise Lynch

**Affiliations:** Trinity College Dublin, Ireland

Thank you for your feedback and taking the time to review our article.

 We have incorporated your feedback (if it was not already included in the latest version) which included adding the correct themes in the abstract, changing words (eg patients to individuals, tools to procedures), replacing PICOS with PICoS and updating the Primsa diagram.

 With reference to changing 'analysis' to 'synthesis', while we understand your reasoning, using Braun and Clarke for qualitative data looks at identifying, analysing, and interpreting patterns of meaning i.e. overall themes within data. Because of this it is a flexible, interpretive method rather than a systematic synthesis of the existing literature. Hence we feel  that analysis should remain and hope that you understand our reasoning.

# Reviewer report: Reviewer response for version 2

**DOI:** 10.21956/hrbopenres.15784.r53786

**Published:** 2026-02-26

**Authors:** Kevin M Korous

**Affiliations:** 1 Medical College of Wisconsin, WI, USA

The author responded thoroughly to my comments and I appreciate that they were open to a discussion. I have no further comments to make.

Are the rationale for, and objectives of, the Systematic Review clearly stated?

No

Is the statistical analysis and its interpretation appropriate?

Yes

Are sufficient details of the methods and analysis provided to allow replication by others?

Partly

Are the conclusions drawn adequately supported by the results presented in the review?

Partly

Reviewer Expertise:

Systematic reviews, meta-analysis, child development, public health, cancer screening

I confirm that I have read this submission and believe that I have an appropriate level of expertise to confirm that it is of an acceptable scientific standard.

# Reviewer report: Reviewer response for version 1

**DOI:** 10.21956/hrbopenres.15576.r48236

**Published:** 2025-08-27

**Authors:** Kevin M Korous

**Affiliations:** 1 Medical College of Wisconsin, WI, USA

Summary

 The authors conducted a literature review to examine which factors contribute to routine cancer screening among individuals with intellectual disabilities. The strength of this study is their focus on a specific, less researched population and I appreciate their use of a literature review to incorporate case studies into the larger picture of evidence. The contribution of this review can be strengthened by addressing the following major and minor limitations.

 Major Limitations

- I suggest that the authors clearly distinguish their paper as a literature review or a systematic review, and subsequently strengthen the rationale. While both methodologies are relatively similar in the overall goal the distinction has implications for evaluating the quality and contribution of the publication. As you will see, many of my comments are from the perspective that it is a systematic review. For instance, I was not sold that a systematic review was necessary aside from the fact that there were little to no published systematic reviews on this topic. If it is intended to be a systematic review, I encourage the authors to articulate the benefits of a systematic review to the gap in literature compared to just a literature review, ultimately helping readers better understand its contribution.
- The authors reported a huge drop in the number of articles included compared to the number identified in their search strategy. While common in systematic reviews, the significant drop does suggest that the search terms could have been more specific. Also, nearly a third of articles were identified through citation searchers, meaning that the search terms could have been more sensitive. Perhaps the authors could use this opportunity to guide future systematic reviews on how to develop sensitive and specific search terms and keywords for identifying research studies on cancer treatment and diagnosis among the population of focus. In other words, what did the authors learn from their search process? Why didn’t the search terms identify the articles retrieved from relevant reviews?
- I recommend that the authors sell the contribution of their review more than they currently do. There is very little discussion on the contribution of the systematic review beyond the studies they included and reviewed. To resolve this issue, the authors could point out the gaps in the literature and what future research or practitioners could do to address such gaps. As the reviewers, the authors are uniquely positioned to address the barriers and advance the literature. There's no discussion of the full picture of evidence, what gaps are present

Minor Limitations

- Abstract does not clearly state the purpose or aim of the literature.
- Add dates of search and any major criteria in the method of the abstract.
- Note how many studies were included in the results of abstract.
- On page 4, under Search Strategy the authors note that relevant reviews were searched. Please cite these reviews.
- It was not clear why the authors exclude reviews and book chapters from the review. If the goal was to examine all factors related to cancer diagnosis and treatment among individual with intellectual disabilities, then reviews and book chapters would have contributed to this conversation. For instance, book chapter may include additional case studies. I recommend that the authors add stronger justification for this exclusion or consider including these types of publications.
- Table 2 was referenced for the list of study characteristics, but Table 2 was the list of keywords and Table 4 had the characteristics. Nonetheless, some factors discussed in the article were not included in Table 4 (e.g., level of ID, measurement method, outcome).
- I recommend addressing the sample size gap between the included studies because without it, readers will overlook the fact that most studies on ID are small, and some are single case studies. What does this tell readers about the state of the literature?
- There were only 3 studies for autonomy, therefore, it was unclear why autonomy was labeled as a major theme across the 35 studies.
- On page 7, living circumstances was described as a both a barrier and a protective factor. It seems odd to label this factor as both unless it is a barrier for some individuals and a protective factor for others; in this case, I recommend that the authors identify the moderating factor in that situation which causes living circumstances to switch its influence. Otherwise, I recommend that the authors stick to one or the other.
- Paragraphs 2 and 3 of the discussion read more like an introduction section, perhaps the authors can move those paragraphs.
- On page 7, “Both the educational programmed and the information leaflet were determined to have a positive effect on individuals’ knowledge and confidence in performing a testicular self-examination.” I recommend that the authors add to this how much these interventions improved knowledge and confidence (e.g., a small shift versus a large jump).
- Did education about concern screening address any of the barriers to obtain screening? Although these themes were separate, it may be helpful to note where these themes overlapped.
- The paragraphs within the first column of page 8 do a really great job at incorporating the findings of the review to the broader research.
- Last paragraph on page 8 brought up ethics as an important factor in cancer treatment and was weaved in some of the themes (e.g., autonomy). I suggest that the authors consider including ethical practices as its own theme as it seems like a special topic that could add to the literature more than the current themes.
- Page 9, regarding limitation “Additionally, cancer diagnosis and treatment may have a geographical influence which may impact study findings.” It wasn’t clear what the geographical limitation was as this was not discussed in the results.
- The final paragraph sells the literature review by suggesting it shows that people with ID should be provided more education, have access to earlier screening, and have more tailored treatment. This conclusion, however, applies to many populations and could be arrived at without a literature review as it is sort of predictable. What is the substantial contribution of this review?
- Page 9 brings up non-“truth-telling”, which caught my attention as a unique contribution and could have been given more attention because it is a behavior/practice that could potentially be changed to improve diagnosis and treatment.

Are the rationale for, and objectives of, the Systematic Review clearly stated?

No

Is the statistical analysis and its interpretation appropriate?

Yes

Are sufficient details of the methods and analysis provided to allow replication by others?

Partly

Are the conclusions drawn adequately supported by the results presented in the review?

Partly

Reviewer Expertise:

Systematic reviews, meta-analysis, child development, public health, cancer screening

I confirm that I have read this submission and believe that I have an appropriate level of expertise to confirm that it is of an acceptable scientific standard, however I have significant reservations, as outlined above.

# Reviewer report: Reviewer response for version 1

**DOI:** 10.21956/hrbopenres.15576.r48116

**Published:** 2025-08-18

**Authors:** Rebecca Hansford

**Affiliations:** 1 Queen's University, Kingston, Canada

Overall

1. The authors should be consistent with wording- e.g., intellectual disability or intellectual disabilities.

Abstract

1. In the background, differences or challenges in providing consent would be more appropriate than the inability to provide consent. Namely, there are individuals with intellectual disability who do provide consent for healthcare-related decisions.

Introduction

1. Para 2: Similar comment regarding inability to provide consent- perhaps challenges in providing consent would be more suitable.

Methods

1. Missing a period after this sentence: “Literature reviews provide a thorough critique and examination of all the evidence available on the topic and facilitate a descriptive analysis and meaningful synthesis of the current available research (Colquhoun et al., 2014)”.
2. Why was a literature review selected rather than a more formal type of review (e.g., scoping, etc.)?
3. The authors should consider extracting information on cancer site from the articles as well- this was conducted somewhat in Table 4, but having a separate extraction item could be useful, and then summarized in the results. 

Results

1. Table 4, which includes all the extracted information (author, year, country, study aim, study design, sample size, age, gender, level of intellectual disability, diagnosis or treatment, measurement method, outcome, summary, conclusions), should potentially be included in the main article rather than the supplementary files.
  1. Table 4- There is a red comma after Armin, rather than black.
  2. 35 studies were included in the PRISMA table, but I only noted 26 listed in Table 4. The authors should update this Table accordingly.
2. The authors should consider adding more details regarding extracted information in a narrative summary at the beginning of the results. For example, how many studies were each type of study design? How many of the studies were quantitative, qualitative, etc? Consequently, there was information about the included studies introduced in the discussion, which could have been mentioned in the results when narratively summarizing the studies.
3. There are two studies that I do not see in the reference list that were published during the study time frame that could be applicable (Brown et al., 2016; Cobigo et al., 2013). I wonder if the authors should add these to the review and reflect on whether it is possible that any other relevant studies were missed.

Brown, H., Plourde, N., Ouellette-Kuntz, H., Vigod, S., & Cobigo, V. (2016). Brief report: cervical cancer screening in women with intellectual and developmental disabilities who have had a pregnancy. *Journal of Intellectual Disability Research*. https://doi.org/10.1111/jir.12225

 Cobigo, V., Ouellette-Kuntz, H., Balogh, R., Leung, F., Lin, E., & Lunksy, Y. (2013). Are cervical and breast cancer screening programmes equitable? The case of women with intellectual and developmental disabilities. *Journal of Intellectual Disability Research*, *57*(5), 478-488. https://doi.org/10.1111/jir.12035

 Discussion

1. As noted in #9, I wonder if some information about studies could have been summarized narratively in the results, allowing for a more focused discussion on barriers, facilitators, etc., in the discussion. One example would be how the authors summarized how adults with intellectual disability were 5 times as likely to never be screened compared to those without intellectual disability (Ishimaru et al., 2022; Lai et al., 2014). While these articles were included in the review, this information was not mentioned until the discussion, and perhaps it would have been more appropriate to note some of these findings from the studies in the results, allowing for a deeper look into why disparities are occurring in the discussion.

Are the rationale for, and objectives of, the Systematic Review clearly stated?

Yes

Is the statistical analysis and its interpretation appropriate?

Yes

Are sufficient details of the methods and analysis provided to allow replication by others?

Yes

Are the conclusions drawn adequately supported by the results presented in the review?

Yes

Reviewer Expertise:

Epidemiology, quantitative research, qualitative research, mixed methods research, intellectual or developmental disabilities, cancer

I confirm that I have read this submission and believe that I have an appropriate level of expertise to confirm that it is of an acceptable scientific standard, however I have significant reservations, as outlined above.

## References

[1] : Brief report: cervical cancer screening in women with intellectual and developmental disabilities who have had a pregnancy. *Journal of Intellectual Disability Research* .2016;60(1) : 10.1111/jir.12225 22-27 10.1111/jir.12225 26359630

[2] : Are cervical and breast cancer screening programmes equitable? The case of women with intellectual and developmental disabilities. *Journal of Intellectual Disability Research* .2013;57(5) : 10.1111/jir.12035 478-488 10.1111/jir.12035 23506206

# Response

**Published:** 2026-01-15

**Authors:** Louise Lynch

**Affiliations:** Trinity College Dublin, Ireland

Thank you both for taking the time to review our article and providing such valuable insights. Responses to each individual comment are included below in ***bold italics***. An updated version of the article will be submitted.

Reviewer 1

 Overall

1. The authors should be consistent with wording- e.g., intellectual disability or intellectual disabilities. ***Amended. Apologies.***

Abstract

1. In the background, differences or challenges in providing consent would be more appropriate than the inability to provide consent. Namely, there are individuals with intellectual disability who do provide consent for healthcare-related decisions. ***Noted and amended.***

Introduction

1. Para 2: Similar comment regarding inability to provide consent- perhaps challenges in providing consent would be more suitable. ***Noted and amended.***

Methods

1. Missing a period after this sentence: “Literature reviews provide a thorough critique and examination of all the evidence available on the topic and facilitate a descriptive analysis and meaningful synthesis of the current available research (Colquhoun et al., 2014)”. ***Added***
2. Why was a literature review selected rather than a more formal type of review (e.g., scoping, etc.)? ***A literature review was selected to facilitate a broad general search on the topic so that results could be synthesised.***
3. The authors should consider extracting information on cancer site from the articles as well- this was conducted somewhat in Table 4, but having a separate extraction item could be useful, and then summarized in the results. ***Apologies we did not feel that this level of detail was needed for this paper as the foucs was on the screening and not the site specific cancers.***

Results

1. Table 4, which includes all the extracted information (author, year, country, study aim, study design, sample size, age, gender, level of intellectual disability, diagnosis or treatment, measurement method, outcome, summary, conclusions), should potentially be included in the main article rather than the supplementary files. ***Table 4 included with relevant information***
  1. Table 4- There is a red comma after Armin, rather than black. ***amended***
  2. 35 studies were included in the PRISMA table, but I only noted 26 listed in Table 4. The authors should update this Table accordingly. ***Amended and two additional articles as recommended were added to bring total to 28.***
2. The authors should consider adding more details regarding extracted information in a narrative summary at the beginning of the results. For example, how many studies were each type of study design? How many of the studies were quantitative, qualitative, etc? Consequently, there was information about the included studies introduced in the discussion, which could have been mentioned in the results when narratively summarizing the studies. ***Amended and moved details as recommended.***
3. There are two studies that I do not see in the reference list that were published during the study time frame that could be applicable (Brown et al., 2016; Cobigo et al., 2013). I wonder if the authors should add these to the review and reflect on whether it is possible that any other relevant studies were missed. ***Apologies these were screened out by mistake. Added now. Others may have been missed and added in as a limitation.***

Discussion

1. As noted in #9, I wonder if some information about studies could have been summarized narratively in the results, allowing for a more focused discussion on barriers, facilitators, etc., in the discussion. One example would be how the authors summarized how adults with intellectual disability were 5 times as likely to never be screened compared to those without intellectual disability (Ishimaru et al., 2022; Lai et al., 2014). While these articles were included in the review, this information was not mentioned until the discussion, and perhaps it would have been more appropriate to note some of these findings from the studies in the results, allowing for a deeper look into why disparities are occurring in the discussion. ***Thank you. The authors have edited the discussion and results with this in mind.***

Reviewer 2

 Summary

 The authors conducted a literature review to examine which factors contribute to routine cancer screening among individuals with intellectual disabilities. The strength of this study is their focus on a specific, less researched population and I appreciate their use of a literature review to incorporate case studies into the larger picture of evidence. The contribution of this review can be strengthened by addressing the following major and minor limitations. ***Thank you.***

 Major Limitations

- I suggest that the authors clearly distinguish their paper as a literature review or a systematic review, and subsequently strengthen the rationale. While both methodologies are relatively similar in the overall goal the distinction has implications for evaluating the quality and contribution of the publication. As you will see, many of my comments are from the perspective that it is a systematic review. For instance, I was not sold that a systematic review was necessary aside from the fact that there were little to no published systematic reviews on this topic. If it is intended to be a systematic review, I encourage the authors to articulate the benefits of a systematic review to the gap in literature compared to just a literature review, ultimately helping readers better understand its contribution. ***Apologies for not making this clearer. This is not a systematic review but a standard literature review. Details have been added in the methodology in the main text and abstract. This is also included in the title. ***
- The authors reported a huge drop in the number of articles included compared to the number identified in their search strategy. While common in systematic reviews, the significant drop does suggest that the search terms could have been more specific. Also, nearly a third of articles were identified through citation searchers, meaning that the search terms could have been more sensitive. Perhaps the authors could use this opportunity to guide future systematic reviews on how to develop sensitive and specific search terms and keywords for identifying research studies on cancer treatment and diagnosis among the population of focus. In other words, what did the authors learn from their search process? Why didn’t the search terms identify the articles retrieved from relevant reviews? ***The authors used broad search terms in the hope of finding all available information as previous experience has shown that potentially limited information is available. The disadvantage of this as you correctly pointed out was a lot of irrelevant information which needed to be screened.  ***
- I recommend that the authors sell the contribution of their review more than they currently do. There is very little discussion on the contribution of the systematic review beyond the studies they included and reviewed. To resolve this issue, the authors could point out the gaps in the literature and what future research or practitioners could do to address such gaps. As the reviewers, the authors are uniquely positioned to address the barriers and advance the literature. There's no discussion of the full picture of evidence, what gaps are present. ***The discussion has been amended and hopefully provides a better reflection of the state of the literature.***

Minor Limitations

- Abstract does not clearly state the purpose or aim of the literature. ***Added***
- Add dates of search and any major criteria in the method of the abstract. ***Added***
- Note how many studies were included in the results of abstract. ***Added***
- On page 4, under Search Strategy the authors note that relevant reviews were searched. Please cite these reviews. ***Apologies these are unavailable.***
- It was not clear why the authors exclude reviews and book chapters from the review. If the goal was to examine all factors related to cancer diagnosis and treatment among individual with intellectual disabilities, then reviews and book chapters would have contributed to this conversation. For instance, book chapter may include additional case studies. I recommend that the authors add stronger justification for this exclusion or consider including these types of publications. ***The authors focussed on original research to reduce bias. However, as you pointed out the relevant reviews were searched for original content.***
- Table 2 was referenced for the list of study characteristics, but Table 2 was the list of keywords and Table 4 had the characteristics. Nonetheless, some factors discussed in the article were not included in Table 4 (e.g., level of ID, measurement method, outcome). ***Apologies. Table numbers changed and amended.***
- I recommend addressing the sample size gap between the included studies because without it, readers will overlook the fact that most studies on ID are small, and some are single case studies. What does this tell readers about the state of the literature? ***A separate limitation section has been added highlighting the overreliance on case studies in this population.***
- There were only 3 studies for autonomy, therefore, it was unclear why autonomy was labeled as a major theme across the 35 studies. ***The theme name has been changed to ethical practice which has meant that two additional studies are not included in this theme.***
- On page 7, living circumstances was described as a both a barrier and a protective factor. It seems odd to label this factor as both unless it is a barrier for some individuals and a protective factor for others; in this case, I recommend that the authors identify the moderating factor in that situation which causes living circumstances to switch its influence. Otherwise, I recommend that the authors stick to one or the other. ***This has been reworded and reordered for clarity.***
- Paragraphs 2 and 3 of the discussion read more like an introduction section, perhaps the authors can move those paragraphs. ***These have been changed and portions moved and deleted.***
- On page 7, “Both the educational programmed and the information leaflet were determined to have a positive effect on individuals’ knowledge and confidence in performing a testicular self-examination.” I recommend that the authors add to this how much these interventions improved knowledge and confidence (e.g., a small shift versus a large jump). ***We reworded the section and this has been deleted.***
- Did education about concern screening address any of the barriers to obtain screening? Although these themes were separate, it may be helpful to note where these themes overlapped. ***Yes, an increase in screening participation was observed but not quantitatively. Modified sentence to include ‘participation’. Shortened the section overall.***
- The paragraphs within the first column of page 8 do a really great job at incorporating the findings of the review to the broader research. ***Thank you.***
- Last paragraph on page 8 brought up ethics as an important factor in cancer treatment and was weaved in some of the themes (e.g., autonomy). I suggest that the authors consider including ethical practices as its own theme as it seems like a special topic that could add to the literature more than the current themes. ***Thank you. This is a really good idea that we did not consider. We have changed the autonomy theme name to ethical practice, which has increased the number of studies in this theme to 5.***
- Page 9, regarding limitation “Additionally, cancer diagnosis and treatment may have a geographical influence which may impact study findings.” It wasn’t clear what the geographical limitation was as this was not discussed in the results. ***Apologies this was in relation to rural living. ‘Residential location’ has been added for clarity.***
- The final paragraph sells the literature review by suggesting it shows that people with ID should be provided more education, have access to earlier screening, and have more tailored treatment. This conclusion, however, applies to many populations and could be arrived at without a literature review as it is sort of predictable. What is the substantial contribution of this review? ***Amended.***
- Page 9 brings up non-“truth-telling”, which caught my attention as a unique contribution and could have been given more attention because it is a behavior/practice that could potentially be changed to improve diagnosis and treatment. ***Thank you. This is a great point and has been added to conclusion.***
